# Predictors of Vitamin D-Containing Supplement Use in the Australian Population and Associations between Dose and Serum 25-Hydroxyvitamin D Concentrations

**DOI:** 10.3390/nu8060356

**Published:** 2016-06-08

**Authors:** Lucinda J. Black, Peter Jacoby, Caryl A. Nowson, Robin M. Daly, Robyn M. Lucas

**Affiliations:** 1 School of Public Health, Curtin University, Bentley 6102, Australia; 2 Telethon Kids Institute, The University of Western Australia, Subiaco 6008, Australia; peter.jacoby@telethonkids.org.au; 3 Institute for Physical Activity and Nutrition (IPAN), School of Exercise and Nutrition Sciences, Deakin University, Geelong 3125, Australia; caryl.nowson@deakin.edu.au (C.A.N.); robin.daly@deakin.edu.au (R.M.D.); 4 National Centre for Epidemiology and Population Health, Research School of Population Health, The Australian National University, Canberra 0200, Australia; robyn.lucas@anu.edu.au

**Keywords:** vitamin D, supplements, 25-hydroxyvitamin D

## Abstract

Despite concerns about vitamin D deficiency in the Australian population, little is known about the prevalence and predictors of vitamin D-containing supplement use. We described the use of vitamin D-containing supplements, and investigated associations between supplemental vitamin D intake and serum 25-hydroxyvitamin D (25(OH)D) concentrations, using a single 24-h dietary recall from the 2011–2013 Australian Health Survey (*n* = 12,153; ages ≥ 2 years). Multiple regression models were used to investigate predictors of vitamin D-containing supplement use in adults, and associations between dose and serum 25(OH)D concentrations/vitamin D sufficiency (≥50 nmol/L), adjusting for potential confounders. The prevalence of vitamin D-containing supplement use was 10%, 6% and 19% in children, adolescents and adults, respectively. Predictors of vitamin D-containing supplement use in adults included being female, advancing age, higher educational attainment, higher socio-economic status, not smoking, and greater physical activity. After adjusting for potential confounders, a 40 IU (1 µg) increase in vitamin D intake from supplements was associated with an increase of 0.41 nmol/L in serum 25(OH)D concentrations (95% CI 0.35, 0.47; *p* < 0.001). However, the prevalence of vitamin D-containing supplement use was generally low in the Australian population, particularly for single vitamin D supplements, with most supplement users obtaining only low levels of vitamin D from other supplement types.

## 1. Introduction

Vitamin D deficiency has traditionally not been considered an important public health issue in Australia due to the abundance of sunlight. However, Australia has the highest skin cancer incidence in the world [1] and public health policies have, therefore, emphasised sun protection practices that limit excessive exposure of the skin to ultraviolet radiation. In a similar timeframe, societal changes, particularly the advent of the personal computer, has meant that many Australians now lead predominantly indoor lifestyles. Findings from the 2011 to 2013 Australian Health Survey showed that 23% of the adult population were vitamin D deficient, defined as serum 25-hydroxyvitamin D (25(OH)D) concentrations <50 nmol/L [2]. The prevalence of vitamin D deficiency varied by ethnicity, and was highest in those born in North Africa, the Middle East and Asia. There is also evidence to suggest a re-emergence of rickets in some Australian children, particularly in recent migrants with dark skin [3]. 

Although the major source of vitamin D in humans is cutaneous synthesis in the presence of sunlight, when sun exposure is limited (e.g., due to sun protection, working indoors, cultural clothing habits, season and weather conditions), dietary sources of vitamin D are required to maintain adequate vitamin D status. Given that natural food sources of vitamin D are limited to meat, fish, eggs, dairy and mushrooms, with vitamin D present mostly in small amounts, supplementation is an alternative for increasing vitamin D intakes and status. Vitamin D supplementation has been shown to substantially increase vitamin D intakes and serum 25(OH)D concentrations in other populations [4,5,6,7,8,9] and the prevalence of supplement use is increasing globally [10,11]. However, very little is known about the prevalence and predictors of vitamin D-containing supplement use in the Australian population. 

The 2011–2013 Australian Health Survey collected comprehensive information on dietary supplement use in a representative sample of the population aged ≥2 years, along with serum 25(OH)D concentrations in those aged ≥12 years measured using internationally-standardised methodology [12]. The aims of this study were to describe the prevalence of vitamin D-containing supplement use in the Australian population, identify independent predictors of vitamin D-containing supplement use in adults, and investigate associations between supplemental vitamin D intake and serum 25(OH)D concentrations.

## 2. Materials and Methods

### 2.1. Study Population

The 2011–2013 Australian Health Survey was the largest, most comprehensive health survey ever conducted in Australia, and aimed to provide a better understanding of the health of people living in Australia [12]. It combined the National Health Survey, the National Nutrition and Physical Activity Survey (NNPAS) and the National Health Measures Survey. The survey excluded residents of non-private dwellings and visitors who were not usual residents of the selected dwelling. Each household contributed either one adult, or one adult plus one child/adolescent.

Core data items were collected for all people in the Australian Health Survey and individuals were then selected to participate either in the National Health Survey (*n* = 20,426), which included detailed health measures and use of medications, or in the NNPAS (*n* = 12,153). The NNPAS included a dietary recall of all food, beverages and supplements for participants aged ≥2 years. Detailed methodology of the NNPAS is available elsewhere [12]. In brief, trained interviewers collected information by face-to-face interview from usual residents of private dwellings in urban and rural areas of Australia. A second interview was conducted by telephone for a subset of participants. To account for possible seasonal effects, the NNPAS was conducted over a 12-month period from May 2011 to June 2012. 

Those aged ≥5 years who participated in the National Health Survey or the NNPAS were invited to participate in the National Health Measures Survey for measurement of nutrient and chronic disease biomarkers (urine test for those aged 5–12 years; blood and urine tests for those aged ≥12 years). Qualified phlebotomists collected fasting and non-fasting blood samples from participants aged ≥12 years (*n* = 10,401) for measurement of biomarkers, including serum 25(OH)D concentrations [12]. No participants were excluded from the National Health Measures Survey due to their health status or life stage.

The interview components of the NHS and NNPAS were conducted under the Census and Statistics Act (CSA) 1905. Ethics approval was sought and gained for the NHMS from the Australian Government Department of Health and Ageing’s Departmental Ethics Committee (Application Number 2/2011). For the NHMS, informed consent was sought from adults and from parents/legal guardians of children through completion of a consent form.

### 2.2. Use of Vitamin D-Containing Supplements

For the purposes of this study, we used data from the first 24-h dietary recall of the NNPAS. Participants were asked: “*Have you taken any dietary supplements in the last 24 h?”* and were encouraged to have in front of them any supplements taken [12]. The type, brand and AUST L number [13] of the supplements were recorded, along with the form (e.g., tablet, capsule) and dose. We determined vitamin D composition data primarily from the Australian Register of Therapeutic Goods, a database of therapeutic goods which can be lawfully supplied in Australia [13]. In some cases, the supplements were not listed in the Australian Register of Therapeutic Goods, either because the product has since been relicensed due to name or formulation change, or the product has been taken off the market. In these cases, vitamin D composition data were obtained from manufacturers by website, telephone call, or email communication. 

Supplements that contained any vitamin D (either added, or inherent in the case of fish liver oil) were considered a vitamin D-containing supplement. A participant who reported using any supplement that contained vitamin D was considered a “vitamin D-containing supplement user”. Vitamin D-containing supplements were classified as single vitamin D supplements, calcium preparations (with added vitamin D), multivitamin/mineral (with added vitamin D), fish oil preparations (with added vitamin D), and fish liver oils (including inherent and added vitamin D).

### 2.3. Analysis of Serum 25-Hydroxyvitamin D Concentrations

The Australian Health Survey is one of eight participating national surveys in the Vitamin D Standardization Program (VDSP), which was established by the National Institute of Health Office of Dietary Supplements in collaboration with the Centers for Disease Control and Prevention and the National Institute for Standards and Technology (NIST). The VDSP aims to internationally standardise the analysis of serum 25(OH)D concentrations [14]. Serum 25(OH)D concentrations for participants aged ≥12 years were measured at the Douglass Hanly Moir (DHM) laboratory by a liquid chromatography-tandem mass spectrometry (LC-MS/MS) method. This laboratory is certified to the standard reference method developed by NIST and Ghent University [14].

### 2.4. Participant Characteristics

We categorised age as follows: 2–4, 5–8, 9–11, 12–13, 14–15, 16–17, 18–30, 31–50, 51–70 and ≥71 years. We defined those aged 2–11 years, 12–17 years and ≥18 years as children, adolescents and adults, respectively. For adult participants, we described the following characteristics: sex, age group, region of birth, State/Territory, education, socioeconomic status, body mass index (BMI) category, physical activity, smoking, alcohol intake, health condition and self-assessed health.

Region of birth was defined as Australia and New Zealand, Europe, Americas, Asia, Africa and the Middle East. Season was defined as: Summer (December–February), Autumn (March–May), Winter (June–August) and Spring (September–November). State/Territory was defined as New South Wales, Victoria, Queensland, South Australia, Western Australia, Tasmania, Northern Territory and Australian Capital Territory. Educational attainment was described as none after secondary school, certificate, Bachelor/Diploma and postgraduate. Socioeconomic status was described by the Socio-Economic Indexes for Areas (SEIFA) 2011 Index of Relative Socio-Economic Disadvantage (IRSD). This is a general socio-economic index that summarises a range of information about the economic and social conditions of people and households within an area, with scores ranging from low (relatively greater disadvantage in general) to high (relative lack of disadvantage in general) [15]. The SEIFA IRSD was categorised into quintiles. 

BMI was calculated from measured weight (kg) and height (m) (weight/height^2^), and adults were categorised as underweight (BMI < 18.5), normal weight (18.5 ≤ BMI < 25.0), overweight (25.0 ≤ BMI < 30.0) or obese (BMI ≥ 30) [12]. Physical activity was defined as low, moderate, or high based on the level of physical activity for fitness, recreation, sport, or walking for transport in the past week [12]. The data items that contributed to this variable included total minutes undertaking moderate exercise/physical activity in the last week; total minutes undertaking vigorous exercise/physical activity in the last week; total minutes walked for fitness, recreation or sport in the last week; and total minutes spent walking for transport in the last week. Each category of physical activity had an intensity factor score (e.g., walking for fitness = 3.5, walking for transport = 3.5, moderate exercise/physical activity = 5 and vigorous exercise/physical activity = 7.5) [12]. The duration of physical activity was multiplied by the intensity factor score. Different levels of exercise/physical activity were defined as: low (no exercise to <800); moderate (800 to 1600, or more than 1600 but with less than 1 h vigorous physical activity); high (>1600 and with 1 h or more of vigorous physical activity).

Smoking was defined as “current smoker”, “ex-smoker” and “never smoked”. Alcohol intake (g/day) was measured by the 24-h dietary recall. Health condition was defined as “yes” or “no” based on whether the participant had ever been told by a medical practitioner that they had one or more of the following conditions (either current or past): diabetes, kidney disease, high cholesterol, high sugar levels, hypertensive disease, ischaemic heart disease, heart failure or other heart disease, cerebrovascular disease, oedema or angina. Self-assessed health was measured as excellent, very good, good, fair or poor.

### 2.5. Statistical Analysis

The prevalence of vitamin D-containing supplement use in the total population was reported by sex, age group and type of supplement used. We determined the proportion of children, adolescents and adults exceeding the Tolerable Upper Intake Level (UL) of vitamin D from supplements on the recording day. The UL is the maximum level of chronic daily intake of a nutrient that is unlikely to pose a risk of adverse health effects to humans [16]. The UL was defined as 2500 IU (62.5 μg) for children aged 1–3 years; 3000 IU (75 μg) for children aged 4–8 years; and 4000 IU (100 μg) for those aged ≥9 years, as per the 2011 Institute of Medicine guidelines [17].

We described characteristics of adult vitamin D-containing supplement users and non-users (*n* = 9435), using number and proportion for categorical variables, and median (interquartile range, IQR) for alcohol intake (g/day). Characteristics of users and non-users of vitamin D-containing supplements were compared using Pearson’s chi-square tests for categorical variables, and the Mann-Whitney U test for alcohol intake.

We used univariate logistic regression to investigate the relationship between vitamin D-containing supplement use in children/adolescents and adults in the same household (*n* = 2634 households; *n* = 5268 individuals). We used multiple logistic regression to investigate the potential predictors of vitamin D-containing supplement use in adults, namely sex, age group, region of birth, State/Territory, season, education, SEIFA, BMI category, physical activity, smoking, alcohol intake, health condition and self-assessed health (*n* = 7751).

We reported the median and IQR for serum 25(OH)D concentrations in adults (stratified by sex and age group) for those taking no vitamin D-containing supplements, and for those taking <400 IU (<10 μg), 400–999 IU (10–24.9 μg), and ≥1000 IU (≥25 μg) of supplemental vitamin D (*n* = 9435). The 400 IU (10 μg) cut-off was chosen to reflect the Estimated Average Requirement of vitamin D for for all ages above one year, assuming minimal sunlight exposure [17], while the 1000 IU (25 μg) cut-off was chosen to reflect the usual daily dose in single vitamin D supplements. 

Using Pearson’s chi-square tests, we compared the major characteristics (sex, age group, region of birth, education and SEIFA) of participants with measured serum 25(OH)D concentrations (*i.e.*, those who agreed to have their blood taken for the measurement of biomarkers, *n* = 3736) *versus* those without measured serum 25(OH)D concentrations (*i.e.*, those who did not agree to have their blood taken for the measurement of biomarkers, *n* = 5699).

We examined associations between vitamin D intake from supplements (as a continuous variable) and serum 25(OH)D concentrations in adults using multiple linear regression models. Models were run unadjusted (*n* = 3736) and adjusted for sex, age group, region of birth, State/Territory, education, SEIFA, BMI category, physical activity, smoking, alcohol intake, health condition and self-assessed health (*n* = 3471). Further, we examined the association between vitamin D intake from supplements and vitamin D sufficiency (defined as ≥50 nmol/L as per the 2011 Institute of Medicine guidelines [17]) in adults using multiple logistic regression, adjusting for the variables above (*n* = 3471). In order to assess whether the associations varied by sex or by season of blood collection, we also stratified these analyses by sex and season.

Analyses were performed using IBM SPSS Statistics Release Version 19.9.9.1 (IBM SPSS Inc., 2010, Chicago, IL, USA) and statistical significance was defined as two-tailed *p* < 0.05.

## 3. Results

### 3.1. Prevalence of Vitamin D-Containing Supplement Use

**Figure 1:**
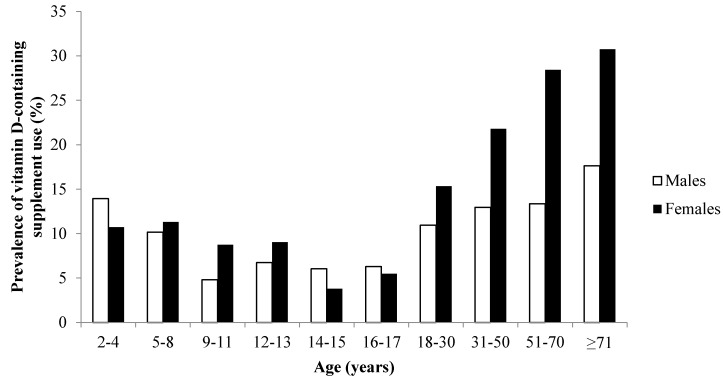
Prevalence of vitamin D-containing supplement use by sex and age group in the Australian population (*n* = 12,153).

Overall, 17% (*n* = 2039) of participants reported using a vitamin D-containing supplement on the recording day: 10% (*n* = 176) of children, 6% (*n* = 63) of adolescents and 19% (*n* = 1800) of adults (Table 1). In adults, there was a clear sex difference in the prevalence of vitamin D-containing supplement use (Figure 1), with 24% of females and 13% of males using vitamin D-containing supplements. The characteristics of adult vitamin D supplement users and non-users are shown in Table 2. 

All single vitamin D supplements used by participants provided a daily vitamin D dose of 1000 IU (25 µg) (Table 3). The median daily dose of vitamin D was 200 IU (5 µg) for multivitamin/mineral, calcium and fish oil preparations; and 80 IU (2 µg) for fish liver oil. The most commonly used vitamin D-containing supplement type was the multivitamin/mineral (males 8%; females 12%) (Table 1). Only 2% of males and 6% of females reported using a single vitamin D supplement, but the prevalence increased with age (7% of males and 15% of females aged ≥71 years). In households with one adult and one child/adolescent participant (*n* = 2634 households), children/adolescents were three times more likely to take a vitamin D-containing supplement if the adult was also a vitamin D-containing supplement user (OR 3.2; 95% CI 2.4, 4.3; *p* < 0.001).

Among the users of vitamin D-containing supplements, 93% of children/adolescents took one vitamin D supplement, while 7% took two vitamin D-containing supplements on the recording day. Among adult users of vitamin D-containing supplements, 86% reported taking one, 13% reported taking two, and 1% reported taking three or more vitamin D-containing supplements on the recording day. Among the users of vitamin D-containing supplements, 47%, 24% and 29% took a daily dose of (<400 IU (<10 µg), 400–999 IU (10–24.9 µg) and (≥1000 IU (≥25 µg), respectively. On the recording day, no children/adolescents exceeded the UL for their age group, and 0.1% of adults (9/9435) exceeded the UL of 4000 IU (100 µg).

### 3.2. Independent Predictors of Vitamin D-Containing Supplement Use

Multiple logistic regression analysis in adults showed that females were more than twice as likely as males to use a vitamin D-containing supplement, and the prevalence of use increased significantly with age group (Table 4). Other predictors of vitamin D-containing supplement use included higher educational attainment, higher socio-economic status, greater physical activity and residential location (highest use in those in Tasmania). Those with at least one health condition and poorer self-assessed health were more likely to use a vitamin D-containing supplement. The prevalence of use was higher in all quintiles of SEIFA compared with the lowest quintile, and in ex-smokers and non-smokers compared with current smokers. There were no significant associations between season or BMI category and vitamin D-containing supplement use.

### 3.3. Associations with Serum 25-Hydroxyvitamin D Concentrations

Participants with measured serum 25(OH)D concentrations were significantly younger (*p* < 0.001) and had a higher level of education (*p* < 0.001) than those without measured 25(OH)D concentrations. There were no significant differences in sex, region of birth and SEIFA in those with and without measured 25(OH)D concentrations.

**Figure 2:**
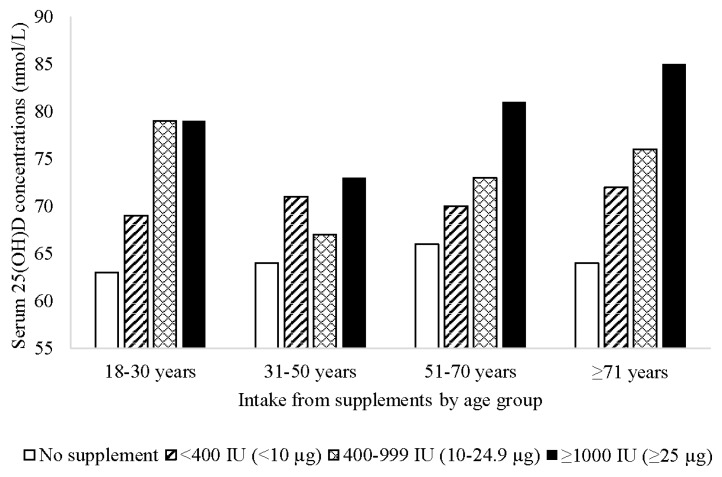
Mean serum 25-hydroxyvitamin D (25(OH)D) concentrations by vitamin D supplement dose and age group among Australian adults (*n* = 3736).

There was a trend towards higher serum 25(OH)D concentrations with increasing dose of supplemental vitamin D intake in adults, which was particularly evident in those aged ≥51 years (Figure 2). In an unadjusted model, vitamin D intake (per 40 IU (1 µg)) from supplements was associated with higher serum 25(OH)D concentrations (coefficient = 0.37; 95% CI 0.31, 0.43; *p* < 0.001). The association remained after adjusting for sex, age group, State/Territory, region of birth, season of interview, education, SEIFA, BMI category, physical activity, smoking, alcohol intake, health condition, and self-assessed health: a 40 IU (1 µg) increase in vitamin D intake from supplements was associated with an increase of 0.41 nmol/L in serum 25(OH)D concentrations (95% CI 0.35, 0.47; *p* < 0.001). When stratified by sex, there was a slightly stronger effect in males (Coefficient = 0.49; 95% CI 0.37, 0.61; *p* < 0.001) than in females (Coefficient = 0.38; 95% CI 0.32, 0.45; *p* < 0.001). When stratified by season, the association between vitamin D intake and serum 25(OH)D concentrations differed marginally depending on season: winter (Coefficient = 0.39; 95% CI 0.27, 0.51; *p* < 0.001); spring (Coefficient = 0.37; 95% CI 0.25, 0.49; *p* < 0.001); summer (Coefficient = 0.37; 95% CI 0.25, 0.49; *p* < 0.001); autumn (Coefficient = 0.48; 95% CI 0.37, 0.59; *p* < 0.001).

Vitamin D intake (per 40 IU (1 µg)) was positively associated with vitamin D sufficiency, defined as ≥50 nmol/L (OR 1.08 per 40 IU (1 µg) increase in supplemental vitamin D; 95% CI 1.06, 1.10; *p* < 0.001) after adjusting for the potential confounders above. When stratified by sex, there was little difference in the association between vitamin D intake and vitamin D status in males (OR 1.07; 95% CI 1.04, 1.11; *p* < 0.001) and females (OR 1.09; 95% CI 1.07, 1.12; *p* < 0.001). When stratified by season, the association between vitamin D intake and vitamin D sufficiency was marginally more pronounced in winter (OR 1.13; 95% CI 1.08, 1.19; *p* < 0.001) than spring (OR 1.08; 95% CI 1.04, 1.12; *p* < 0.001), summer (OR 1.06; 95% CI 1.02, 1.11; *p* = 0.003) and autumn (OR 1.09; 95% CI 1.05, 1.13; *p* < 0.001). 

## 4. Discussion

This study provides new information on the prevalence and predictors of vitamin D-containing supplement use in the Australian population, based on nationally representative data. We found that 10% of children, 6% of adolescents and 19% of adults reported using a vitamin D-containing supplement. In accordance with other studies [6,9,18], the prevalence of vitamin D-containing supplement use in adults was higher in females than males, with the highest prevalence of use being in older females. The prevalence of vitamin D-containing supplement use reported in the United States (US) and Canada is substantially higher than that reported in our study. For example, in the 2005–2006 National Health and Nutrition Examination Survey (NHANES), vitamin D supplement use in the US ranged from 16% to 43% in children/adolescents, and from 21% to 49% in adults, depending on sex and age group [13]. In the 2007–2009 Canadian Health Measures Survey (CHMS), 31% of 6–79 year olds reported using vitamin D-containing supplements, with the majority of users being female [9]. Both the NHANES and CHMS employed questionnaires that captured vitamin D-containing supplement use in the previous month, as opposed to the single 24-h dietary recall used in our study. Nevertheless, there may genuinely be a higher prevalence of vitamin D-containing supplement use in the US and Canada compared with Australia, since the importance of dietary vitamin D is emphasised in these countries, with vitamin D food fortification also widely practiced. In addition, Canada and parts of the US lie at high latitudes where cutaneous synthesis of vitamin D is limited during winter months, necessitating diet and supplements as an alternative source of vitamin D. 

In Ireland, vitamin D-containing supplements were used by 16% of the adult population based on data derived from a 4-day food record from the 2009 National Adult Nutrition Survey [8]. This is similar to the prevalence observed in our study of Australians. However, in a 7-day food record from the 2004 Irish Children’s and 2006 Irish Teens’ Food Consumption Surveys, vitamin D-containing supplement use was 21% in 5–8 year olds, 16% in 9–12 year olds and 15% in 13–17 year olds [4], which is substantially higher than in our study. This higher prevalence of supplement use amongst children and adolescents may again reflect an awareness of the importance of vitamin D supplementation in a country of high latitude and inclement weather conditions.

Overall, relatively few participants in our study used a single vitamin D supplement—the most commonly used vitamin D-containing supplement type was the multivitamin/mineral. Users of multivitamin/minerals, fish oil and fish liver oil are likely to be taking these supplements for nutritional qualities other than vitamin D. Low dose vitamin D-containing supplements, such as those found in most multivitamin/mineral preparations, are not sufficient to alleviate vitamin D deficiency and may not provide health benefits such as prevention of falls in the elderly. In our study, the highest prevalence of vitamin D-containing supplement use and single vitamin D supplement use was in females aged ≥51 years, who are the group with the greatest risk of osteoporosis with increasing age. The greater use of single vitamin D supplements in this population group may reflect a response to recommendations from health professionals to ensure adequate vitamin D status, particularly for reducing risk of falls and fractures. Although nine adults exceeded the UL as defined by the Institute of Medicine as 4000 IU (100 µg), some studies have shown no adverse effects with higher daily doses e.g., 6400 IU (160 µg) [19] and 10,000 IU (250 µg) [17].

Higher level of education and higher SEIFA were associated with vitamin D-containing supplement use in the Australian population. Such a pattern is consistent with that reported in other studies [9,18]. This pattern is also apparent in overall dietary supplement use in some countries, including Canada [20] and the US [21,22]. Focus groups in Canada have shown that cost, accessibility, knowledge about what to purchase and lack of information are the most common barriers to supplement use [23]. In Australia, vitamin D supplements are available over-the-counter, and there are no subsidies available for low-income earners who are recommended vitamin D supplements by their general practitioner. 

As with other studies, we found a trend towards higher prevalence of vitamin D-containing supplement use in those who were most physically active. Higher overall dietary supplement use in those who are physically active has been reported in other studies [22,24] and may be due to the perceived benefits of dietary supplements on performance [25] or more health-conscious behaviours in general [26]. Although we found no association between BMI category and vitamin D-containing supplement use, there is evidence of lower serum 25(OH)D concentrations with increased adiposity [27,28,29].

We found that adults born in Asia were more likely to use vitamin D-containing supplements compared with those born in Australia and New Zealand. Despite the fact that vitamin D deficiency is higher during winter months in most regions of Australia [2], we found no seasonal differences in the prevalence of vitamin D-containing supplement use. The use of vitamin D-containing supplements was least likely among adults living in the Northern Territory and most likely among adults living in Tasmania. This is in line with the relatively low risk of vitamin D deficiency in those residing closer compared to further from the equator (e.g., Northern Territory compared to Tasmania) [2]. 

As expected, vitamin D intake from supplements was independently associated with higher serum 25(OH)D concentrations and with greater likelihood of vitamin D sufficiency (≥50 nmol/L), a finding supported by population-based studies in Canada [7,9]. Our results show that a 1000 IU (25 µg) dose of supplemental vitamin D (the standard daily dose in a single vitamin D supplement) equates to an average increase in serum 25(OH)D concentrations of 10 nmol/L, and that those who use a single vitamin D-containing supplement are significantly more likely to be vitamin D sufficient than those who do not take a supplement. It should be noted, however, that although the Institute of Medicine suggest that practically all persons are sufficient at serum 25(OH)D concentrations of ≥50 nmol/L, the value of >75 nmol/L has been proposed by others [30]. Furthermore, there is some evidence to suggest that concentrations of 75–100 nmol/L are required for optimal health, particularly in relation to non-skeletal health outcomes [31].

A strength of our study was the availability of comprehensive dietary and demographic data from a nationally representative survey. Supplement intake data were collected directly from participants using face-to-face interview and participants were encouraged to have in front of them any supplements taken. Vitamin D composition data for each product were transcribed from the Australian Register of Therapeutic Goods or from manufacturers’ information, ensuring that composition data were accurate at brand and product level. It should be noted, however, that this study was based on supplement intake data from a single 24-h dietary recall, and use on any particular day may not reflect consistent and long-term use of supplements. In addition, we found that adult participants who provided a blood sample for measurement of serum 25(OH)D concentrations were younger and had a higher level of education than adult participants who did not provide a blood sample. For this reason, associations between supplement dose and serum 25(OH)D concentrations/vitamin D status may not be generalisable to the total population.

## 5. Conclusions

To our knowledge, this is the first detailed investigation of vitamin D-containing supplement use in a nationally-representative sample of the Australian population. Consistent with international populations, the predictors of vitamin D-containing supplement use include higher socio-economic status, higher educational attainment and higher levels of physical activity. We found that the use of vitamin D-containing supplements was independently associated with higher serum 25(OH)D concentrations and the likelihood of vitamin D sufficiency. However, the prevalence of vitamin D-containing supplement use was generally low in the Australian population, particularly for single vitamin D supplements, with most supplement users obtaining only low levels of vitamin D from other supplement types. Promoting safe levels of sun exposure for optimal health, and introducing food-based strategies to increase vitamin D in the food supply, are likely to include a larger section of the population and may be more effective in reducing the prevalence of vitamin D deficiency in Australia.

## Figures and Tables

**Table 1:** Prevalence of vitamin D-containing supplement use in the Australian population (*n* = 12,153) by sex, age group and supplement type.

	2–11 Years	12–17 Years	18–30 Years	31–50 Years	51–70 Years	≥71 Years	≥2 Years	≥18 Years
	*n* (%)	*n* (%)	*n* (%)	*n* (%)	*n* (%)	*n* (%)	*n* (%)	*n* (%)
**Total**								
All types	176 (10.3)	63 (6.3)	224 (13.3)	629 (17.6)	624 (21.5)	323 (25.3)	2039 (16.8)	1800 (19.1)
Single vitamin D	1 (0.1)	5 (0.5)	26 (1.5)	104 (2.9)	218 (7.5)	147 (11.5)	501 (4.1)	495 (5.2)
Calcium ^1^	17 (1.0)	6 (0.6)	5 (0.3)	67 (1.9)	157 (5.4)	72 (5.6)	324 (2.7)	301 (3.2)
MVMM ^1^	155 (9.1)	50 (5.0)	179 (10.6)	460 (12.9)	300 (10.3)	128 (10.0)	1272 (10.5)	1067 (11.3)
Fish oil ^1^	7 (0.4)	7 (0.7)	26 (1.5)	50 (1.4)	42 (1.4)	15 (1.2)	147 (1.2)	133 (1.4)
Fish liver oil ^2^	4 (0.2)	0 (0.0)	1 (0.1)	10 (0.3)	16 (0.6)	16 (1.3)	47 (0.4)	43 (0.5)
**Males**								
All types	87 (10.2)	33 (6.4)	86 (10.9)	216 (12.9)	179 (13.3)	94 (17.6)	695 (12.2)	575 (13.3)
Single vitamin D	1 (0.1)	1 (0.2)	6 (0.8)	27 (1.6)	51 (3.8)	36 (6.8)	122 (2.1)	120 (2.8)
Calcium ^1^	9 (1.1)	3 (0.6)	3 (0.4)	14 (0.8)	19 (1.4)	11 (2.1)	59 (1.0)	47 (1.1)
MVMM ^1^	75 (8.8)	26 (5.0)	68 (8.7)	161 (9.6)	99 (7.4)	45 (8.4)	474 (8.3)	373 (8.6)
Fish oil ^1^	3 (0.4)	4 (0.8)	14 (1.8)	28 (1.7)	19 (1.4)	6 (1.1)	74 (1.3)	67 (1.5)
Fish liver oil ^2^	2 (0.2)	0 (0.0)	0 (0.0)	5 (0.3)	4 (0.3)	6 (1.1)	17 (0.3)	15 (0.3)
**Females**								
All types	89 (10.4)	30 (6.1)	138 (15.3)	413 (21.8)	445 (28.4)	229 (30.7)	1344 (20.8)	1225 (24.0)
Single vitamin D	0 (0.0)	4 (0.8)	20 (2.2)	77 (4.1)	167 (10.7)	111 (14.9)	379 (5.9)	375 (7.3)
Calcium ^1^	8 (0.9)	3 (0.6)	2 (0.2)	53 (2.8)	138 (8.8)	61 (8.2)	265 (4.1)	254 (5.0)
MVMM ^1^	80 (9.3)	24 (4.9)	111 (12.3)	299 (15.8)	201 (12.8)	83 (11.1)	798 (12.4)	694 (13.6)
Fish oil ^1^	4 (0.5)	3 (0.6)	12 (1.3)	22 (1.2)	23 (1.5)	9 (1.2)	73 (1.1)	66 (1.3)
Fish liver oil ^2^	2 (0.2)	0 (0.0)	1 (0.1)	5 (0.3)	12 (0.8)	10 (1.3)	30 (0.5)	28 (0.5)

^1^ With added vitamin D; ^2^ Inherent vitamin D with/without added vitamin D; MVMM, multivitamin-multimineral.

**Table 2:** Characteristics of vitamin D-containing supplement users and non-users among Australian adults (*n* = 9435).

	*n*	% Across Rows	*n*	% Across Rows	*p*
	Vitamin D-Containing Supplement User		Vitamin D-Containing Supplement Non-User		
**Sex**	***1800***		***7635***	****	<0.001
Male	*575*	13	*3754*	87	
Female	*1225*	24	*3881*	76	
**Age Group**	***1800***		***7635***		<0.001
18–30 years	*224*	13	*1462*	87	
31–50 years	*629*	18	*2936*	82	
51–70 years	*624*	21	*2282*	79	
≥71 years	*323*	25	*955*	75	
**Region of birth**	***1800***		***7635***		0.009
Australia and New Zealand	*1277*	18	*5729*	82	
Europe	*275*	21	*1017*	79	
Americas	*26*	21	*98*	79	
Asia	*166*	22	*574*	78	
Africa and Middle East	*56*	21	*217*	79	
**State/Territory**	***1800***		***7635***		<0.001
New South Wales	*312*	19	*1345*	81	
Victoria	*276*	20	*1085*	80	
Queensland	*271*	18	*1239*	82	
South Australia	*248*	21	*952*	79	
Western Australia	*258*	19	*1067*	81	
Tasmania	*205*	21	*791*	79	
Northern Territory	*68*	12	*507*	88	
Australian Capital Territory	*162*	20	*649*	80	
**Education**	***1767***		***7524***		<0.001
None after school	*601*	16	*3103*	84	
Certificate	*366*	17	*1849*	83	
Bachelor/Diploma	*597*	23	*2005*	77	
Postgraduate	*203*	26	*567*	74	
**SEIFA**	***1800***		***7635***		<0.001
Lowest 20%	*270*	15	*1508*	85	
Second quintile	*360*	18	*1601*	82	
Third quintile	*397*	21	*1476*	79	
Fourth quintile	*379*	20	*1537*	80	
Highest 20%	*394*	21	*1513*	79	
**BMI category**	***1527***		***6431***		0.491
Underweight	*24*	20	*97*	80	
Normal weight	*544*	20	*2192*	80	
Overweight	*559*	19	*2339*	81	
Obese	*400*	18	*1803*	82	
**Physical activity**	***1777***		***7551***		0.218
Low	*1001*	18	*4425*	82	
Moderate	*512*	20	*2062*	80	
High	*264*	20	*1064*	80	
**Smoking**	***1800***	****	***7635***		<0.001
Current smoker	*197*	11	*1588*	89	
Ex-smoker	*660*	21	*2417*	79	
Never smoked	*943*	21	*3630*	79	
**Alcohol (g/day) (median, IQR)**	***1800***	0.0 (14.4)	***7635***	0.0 (16.3)	0.006
**Health condition**	***1800***		***7635***		<0.001
Yes	*912*	22	*3155*	78	
No	*888*	17	*4480*	83	
**Self-assessed health**	***1800***		***7635***		0.738
Excellent	*286*	19	*1251*	81	
Very good	*647*	19	*2676*	81	
Good	*556*	19	*2409*	81	
Fair	*217*	19	*946*	81	
Poor	*94*	21	*353*	79	

IQR, interquartile range.

**Table 3:** Description of vitamin D dose (IU (µg)/day) in vitamin D-containing supplements used by survey participants.

Type of Supplement	Minimum	Maximum	Median	IQR
Single vitamin D	1000 (25.0)	1000 (25.0)	1000 (25.0)	0 (0.0)
Multivitamin/mineral	4 (0.1)	1000 (25.0)	200 (5.0)	224 (5.6)
Calcium preparation	5.2 (1.3)	1000 (25.0)	200 (5.0)	200 (5.0)
Fish oil preparation	4 (0.1)	1000 (25.0)	200 (5.0)	264 (6.6)
Fish liver oil	8 (0.2)	252 (6.3)	80 (2.0)	40 (1.0)

IQR, interquartile range.

**Table 4:** Adjusted logistic regression model investigating independent predictors of vitamin D-containing supplement use in Australian adults (*n* = 7751).

	Adjusted OR (95% CI)	*p*
**Sex (female *vs.* male)**	2.05 (1.80, 2.33)	<0.001
**Age group**		<0.001
18–30 years	Reference category	
31–50 years	1.36 (1.12, 1.64)	0.002
51–70 years	2.00 (1.60, 2.41)	<0.001
71 years and over	2.40 (1.88, 3.05)	<0.001
**Region of birth**		0.088
Australia and New Zealand	Reference category	
Europe	0.97 (0.82, 1.15)	0.705
Americas	1.21 (0.75, 1.94)	0.435
Asia	1.26 (1.01, 1.57)	0.044
Africa and Middle East	1.38 (1.00, 1.92)	0.052
**State/Territory**		0.002
New South Wales	Reference category	
Victoria	1.19 (0.97, 1.46)	0.104
Queensland	0.99 (0.81, 1.22)	0.949
South Australia	1.22 (0.98, 1.50)	0.074
Western Australia	1.11 (0.90, 1.36)	0.344
Tasmania	1.30 (1.04, 1.63)	0.023
Northern Territory	0.64 (0.46, 0.88)	0.007
Australian Capital Territory	1.10 (0.86, 1.40)	0.460
**Season**		0.415
Summer	Reference category	
Autumn	1.12 (0.96, 1.31)	0.151
Winter	1.10 (0.93, 1.30)	0.267
Spring	1.14 (0.96, 1.34)	0.150
**Education**		<0.001
None after school	Reference category	
Certificate	1.27 (1.08, 1.50)	0.004
Bachelor/Diploma	1.63 (1.40, 1.89)	<0.001
Postgraduate	1.87 (1.44, 2.42)	<0.001
**SEIFA**		<0.001
Lowest 20%	Reference category	
Second quintile	1.27 (1.04, 1.55)	0.017
Third quintile	1.65 (1.35, 2.01)	<0.001
Fourth quintile	1.31 (1.07, 1.61)	0.009
Highest 20%	1.31 (1.06, 1.61)	0.012
**BMI category**		0.160
Normal weight	Reference category	
Overweight	0.99 (0.86, 1.14)	0.876
Obese	0.85 (0.73, 1.00)	0.050
Underweight	1.12 (0.70, 1.81)	0.638
**Physical activity**		0.006
Low	Reference category	
Moderate	1.09 (0.95, 1.25)	0.205
High	1.34 (1.12, 1.61)	0.002
**Smoking**		<0.001
Current smoker	Reference category	
Ex-smoker	1.73 (1.42, 2.10)	<0.001
Never smoked	1.58 (1.30, 1.91)	<0.001
**Alcohol (g/day)**	1.00 (1.00, 1.00)	0.802
**Health condition (yes v no)**	1.24 (1.09 1.42)	0.001
**Self-assessed health**		0.019
Excellent	Reference category	
Very good	1.22 (1.02, 1.46)	0.032
Good	1.31 (1.08, 1.59)	0.005
Fair	1.30 (1.03, 1.66)	0.031
Poor	1.64 (1.19, 2.26)	0.003

## Abbreviations

The following abbreviations are used in this manuscript:

25(OH)D	25-hydroxyvitamin D
NNPAS	National Nutrition and Physical Activity Survey
SEIFA	Socio-Economic Indexes for Areas
UL	Tolerable Upper Intake Level
